# Quantitative evaluation of China's adolescent physical activity policies: a PMC Index model approach

**DOI:** 10.3389/fpubh.2025.1625225

**Published:** 2025-07-31

**Authors:** Xinzhu Huang, Changsheng Zhang, Jiang Li, Xianming Ding

**Affiliations:** ^1^Chengdu Sport University, Chengdu, Sichuan, China; ^2^College of Sports and Health Management of Chongqing University of Education, Chongqing, China; ^3^College of Sports Science, Jishou University, Jishou, Hunan, China

**Keywords:** adolescent physical activity policies, PMC-Index model, policy evaluation, text mining, public health

## Abstract

**Background:**

China has promulgated a series of policies to address the health crisis among adolescents resulting from physical inactivity. These policies aim to promote physical activity among Chinese Adolescent and improving health. As institutional mechanisms for building social consensus and providing strategic guidance, evaluating and optimizing these policies to enhance their efficacy represents a critical imperative.

**Method:**

This paper employs the PMC-Index model to conduct text mining on 9 adolescent Physical fitness policies implemented in China since the inception of the new century. This paper first constructs PMC-Index model, then conducts quantitative analysis on the text, and finally calculates the scores of nine policies in various dimensions to analyze the advantages and disadvantages of the policies. In addition, this paper performs visual analysis by drawing a curved surface diagram.

**Result:**

The nine adolescent sports health promotion policies in China yield an average score of 6.83, indicating a generally sound status overall. However, deficiencies exist in supervising policy implementation, coordinating stakeholder responsibilities, and ensuring policy safeguards.

**Conclusions:**

The results indicate that China should strengthen the application of youth sports supply-side policy instruments in the future, clarify the division of responsibilities among schools, families and communities, improve the reward and punishment mechanism of policy implementation, and effectively improve the effectiveness of youth Physical fitness policies.

## 1 Introduction

It is clear that adolescent health is critical to personal growth, the well-being of families and the future of nations. It is an engine for a healthier and more sustainable social transformation (1). However, according to the 2022 World Health Organization's Global Health report, 81% of adolescents currently do not meet WHO recommended levels of physical activity, leading to a surge in non-communicable diseases (NCDs) (2). In response to this worldwide health crisis, many countries around the world have promulgated and implemented adolescent physical activity promotion policies. The aim of these policies is to improve the Physical fitness of adolescents. For example, the policies promulgated by Britain include *Creating a Sporting Habit for Life: A New Youth Sport Strategy, Child and Adolescent Health Promotion Strategy, Daily Mile*, et al. The US has introduced corresponding policies including *The National Youth Sports Strategy* and *Comprehensive School Physical Activity Program (CSPAP)*. Canada has enacted *Canadian Sport for Life* (3–6). These countries aim to guide the regions and schools to pay attention to the Physical fitness of teenagers through special policies, and provide environmental conditions for the participation of teenagers in sports (7, 8).

As a country with Confucian culture in Asia, China has always attached great importance to education, and students' high achievement in study is the common expectation. This ideological tradition has made China's education environment highly competitive. Driven by the upward logic of exam-oriented culture, schools and parents devote a lot of time and energy to students' cultural knowledge learning, but ignore physical exercise. This situation has led to a serious lack of physical exercise among young people in China, and a high rate of obesity and myopia (9). Through a top-down policy system, the Chinese government has also incorporated the work on adolescents‘ Physical fitness into a comprehensive framework encompassing strategic planning, coordinated implementation, and binding requirements to systematically promote the work on adolescents' Physical fitness and solve the problems of adolescents' Physical fitness. Notably, the Chinese government has institutionalized established the 1-h system of intramural physical exercise through “two exercises, two lessons, and two activities” (a structured framework for school-based physical activity), and established a dynamic monitoring mechanism with big data of students' Physical fitness testing, providing a systematic system plan for solving the imbalance between intellectual education and physical education structure (10).

Entering the new century, China's economic and social transformation is accelerating, but young people are facing the double challenges of intense academic pressure and static lifestyle superimposed (11). It is worth noting that despite China's continuous national policy supply, the country's youth physical fitness level is still a shortcoming of comprehensive literacy, and there is a significant gap with the comprehensive development talent reserve required by the Chinese government. In this context, scientific assessment of the quality of China's existing policies and accurate identification of optimization paths have become key tasks to improve policy effectiveness.

Therefore, we first used ROST CM software to mine and cluster adolescent Physical fitness promotion policy texts since 2000, then constructed PMC-Index model and systematically evaluated nine of the most influential policies through quantitative indicators to reveal the effectiveness, content completeness and practical fit of existing policy texts. Then the targeted optimization suggestions are formed.

## 2 Literature review

### 2.1 Policy research on adolescent physical fitness and health

Adolescent physical activity policies refer to institutional arrangements that aim to improve youth health levels through exercise-based interventions, encompassing strategic decisions, administrative mechanisms, and supervisory guarantees (12). After reviewing the literature, we roughly found that existing studies on adolescent physical activity policies mainly focus on three dimensions: policy evolution, policy components, and policy implementation. First, a chronological analysis of adolescent physical activity policies has been conducted to identify their evolutionary patterns (13, 14). For example, in China, the policy trajectory has undergone a transformation from initial institutionalization, standardization and legalization, to consolidation and socialization. This process reflects a governance paradigm shift from “Strengthening Physical Fitness” to “Integration of Sports and Education (15–17).” Second, scholars have explored key policy components such as national recommendations, national goals, surveillance systems, and public education (18). These studies analyze how elements including physical activity promotion, service provision, and supportive mechanisms within policies affect the enhancement of adolescent physical fitness (19, 20). Third, research has examined various forms of policy implementation, including symbolic implementation, selective implementation, and additive implementation, in order to assess their effects on increasing physical activity and reducing sedentary behavior (21–23). These reflections have also drawn attention to limitations within the policies themselves. Although prior studies have made valuable contributions to understanding adolescent physical activity policies, several limitations remain: (1) Limited analytical scope: many papers tend to interpret policy background, intent, or content from a narrative perspective, with a strong degree of subjectivity. (2) Insufficient critical reflection: while developmental patterns are often analyzed from a temporal perspective, there is a lack of in-depth critique regarding policy shortcomings. (3) Fragmented evaluation approaches: despite the use of data analysis or surveys based on policy texts, there is a scarcity of studies employing unified and multidimensional indicators to conduct systematic quantitative evaluation. Consequently, in-depth analysis that can accurately identify the strengths and weaknesses of policies remains insufficient.

### 2.2 Applications of the PMC-Index model

Quantitative research on policy texts primarily draws upon the methods and knowledge of disciplines such as statistics and econometrics to conduct text mining, content analysis, and functional identification, thereby enabling comprehensive evaluation of policies (24). The policy modeling consistency (PMC) index model is one such method developed from a quantitative policy evaluation perspective. It constructs a unified evaluation indicator system based on the textual elements of policies, aiming to provide a multidimensional and systematic assessment of policy quality (25). First proposed by Ruiz Estrada (26), the PMC-Index model has rapidly gained traction due to its analytical advantages and has since been widely applied across various policy domains. It is now considered a cutting-edge and popular tool in the field of policy evaluation. For instance, Li et al. (27) conducted a quantitative evaluation of science education policies in primary and secondary schools in China during the 14th Five-Year Plan period. Their results highlighted limitations in policy nature, policy perspectives, and policy focus, and provided targeted recommendations for policy optimization. Similarly, Zhang et al. (28) evaluated 10 highly representative data security policies issued between 2016 and 2023, noting deficiencies in the practicality, orientation, and coordination of policy tools, and advocated for enhancing the utility value of hybrid policy instruments. In addition, the PMC-Index model has been extensively applied in the evaluation of policies supporting traditional Chinese medicine (29–31), clinical research policies (32), policies for promoting the high-quality development of provincial-level public hospitals (33), and the “Healthy China” initiative (34). These studies demonstrate the model's broad applicability and strong objectivity, offering valuable insights for both theoretical research and practical policy making.

## 3 Materials and methods

### 3.1 Technical roadmap

**Figure 1:**
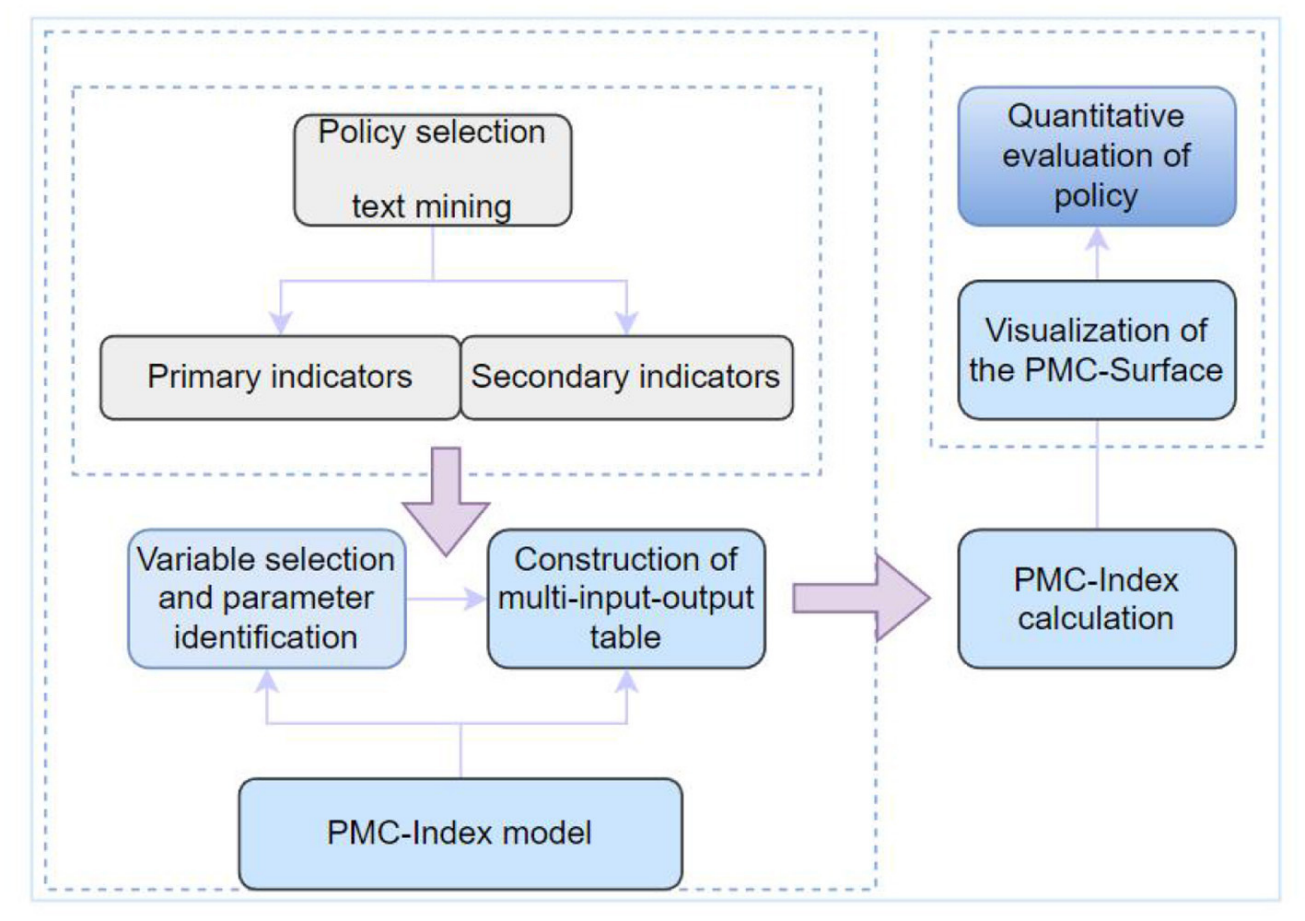
Technical roadmap of the research.

The construction of the PMC-Index model involves four main steps: variable selection and parameter identification; construction of the multi-input-output table; calculation of the PMC-Index; and drawing of PMC surface diagram (35, 36). To obtain more accurate evaluation indicators, we have enhanced the mining of policy texts, thereby aligning the evaluation more closely with Physical fitness policies (Figure 1).

### 3.2 Data collection

In the process of collecting policies related to adolescent Physical fitness promotion, this study adopted the following retrieval strategy to ensure both comprehensiveness and accuracy of the policy texts. First, keywords such as “physical fitness,” “adolescent sports,” “school sports,” and “physical and mental health” were used to search for documents across multiple platforms, including the “Government Documents” database on CNKI (China National Knowledge Infrastructure) and the official websites of relevant central government agencies (e.g., the State Council, General Administration of Sport of China, Ministry of Education, and National Health Commission). A total of 282 policy documents were obtained from this initial search. Second, the documents were filtered and de-duplicated according to the following criteria: (1) publication dates ranging from January 2000 to December 2024; (2) policies targeting adolescents or students; (3) policies promoting physical fitness through physical activity. After applying these filters, a total of 40 policy documents were retained for further analysis. Third, the policy texts were imported into ROSTCM6.0 software for preprocessing through data cleaning. Nouns and general terms with limited semantic value—such as “student,” “development,” and “work”—were filtered out. Subsequently, semantically similar terms, such as “sports” and “exercise” or “stadium” and “playground,” were merged to generate the high-frequency term list (Table 1).

**Table 1:** High-frequency keywords in adolescent physical activity policies.

**No**.	**Keyword**	**Frequency**	**No**.	**Keyword**	**Frequency**	**No**.	**Keyword**	**Frequency**	**No**.	**Keyword**	**Frequency**
1	Sport	4269	11	Standard	488	21	Guarantee	345	31	Resource	251
2	Health	1753	12	Teacher	414	22	Innovation	334	32	Nutrition	245
3	Education	1339	13	Physical fitness	389	23	Government	334	33	Training	245
4	School	1130	14	Competition	389	24	Sports venue	297	34	National fitness	236
5	Society	769	15	Primary school	388	25	Culture	275	35	Quality	229
6	Service	763	16	Cultivation	385	26	Football	272	36	Specification	228
7	Physical activity	754	17	Teaching	375	27	Competency	270	37	Knowledge	227
8	Management	686	18	Hygiene	365	28	Training (skill)	268	38	Environment	218
9	Reform	584	19	Guidance	363	29	Medical care	259	39	Technology	202
10	Evaluation	509	20	Facility	349	30	Public	253	40	Monitoring	193

**Figure 2:**
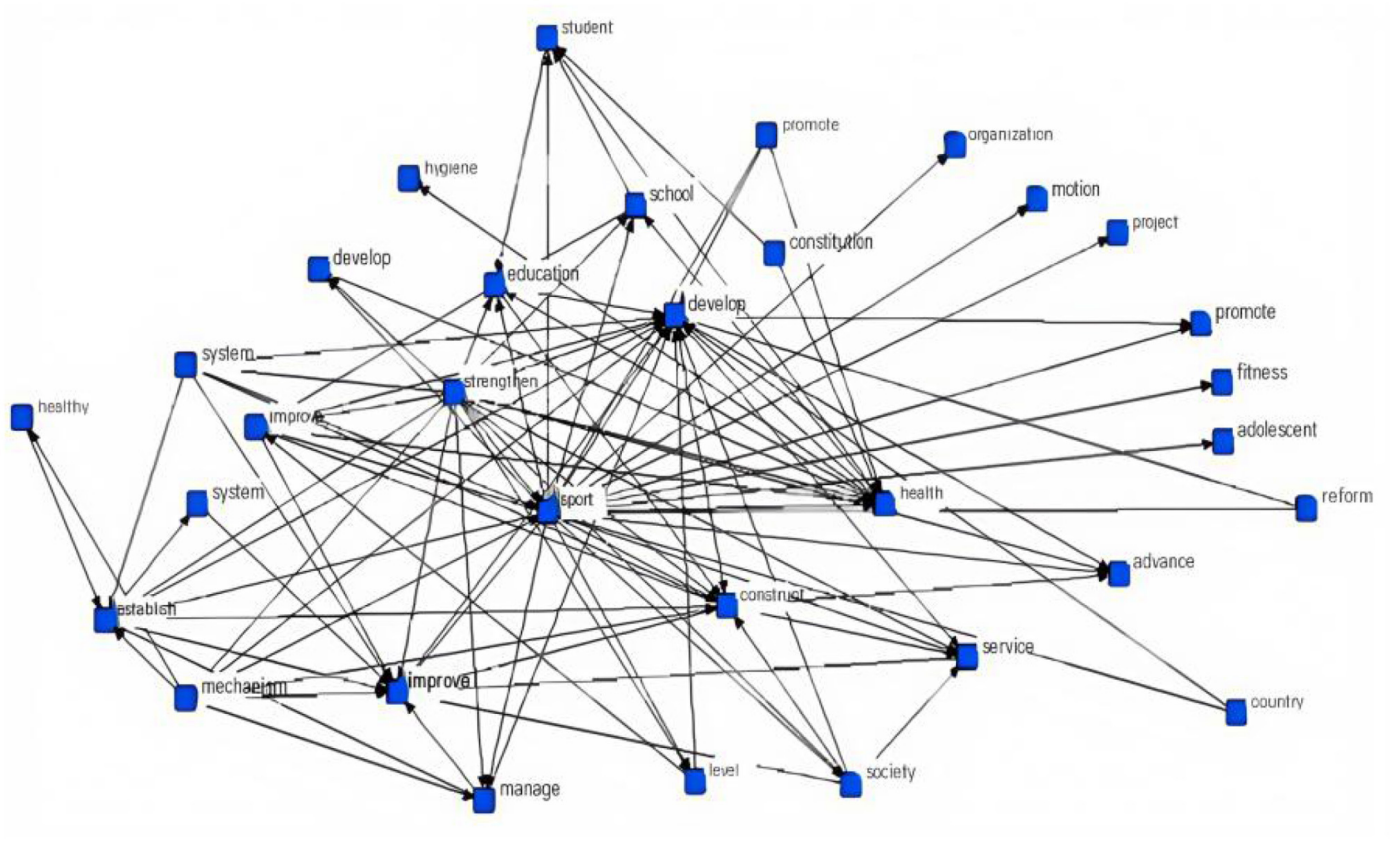
Semantic co-occurrence network of adolescent physical activity promotion policies.

High-frequency terms with a frequency >150 from the adolescent Physical fitness promotion policy corpus were retained. Using the “semantic network co-occurrence” function of the software, these high-frequency thematic terms were connected in the form of a frequency matrix (Figure 2). The nodes “sports” and “health” emerged as the most prominent, occupying the core positions within the graph. The terms most frequently connected to them—such as “school” and “education”—highlight the central policy logic: promoting adolescent physical activity primarily through school-based education to foster health and holistic development. Furthermore, the frequent appearance of terms like “management,” “reform,” “evaluation,” and “supervision” underscores the government's consistent prioritization of adolescent health, emphasizing policy implementation through enhanced governance and oversight mechanisms. In addition, terms such as “teachers,” “facilities,” “stadiums,” and “competitions” reflect the infrastructural and human resource requirements essential for delivering physical activity programs. Notably, terms like “public health” and “public services” have become increasingly prevalent in recent years, indicating a paradigm shift—adolescent Physical fitness promotion is no longer confined to the education sector, but is gradually being integrated into the broader public service system, necessitating joint efforts from schools, community organizations, and sports clubs.

First, based on the frequency statistics and the semantic network of high-frequency terms, and following the principles of broad policy coverage, high thematic relevance, and strong policy influence, 12 policies were initially selected from the original pool of forty. Second, the expert consultation method was applied. Eight experts with extensive experience in adolescent physical fitness and a strong sense of responsibility and enthusiasm for school sports were invited to evaluate whether each policy qualified as a “representative policy”. In the first round of evaluation, three policies did not receive support from a majority of the experts and were therefore excluded. Finally, a second round of consultation was conducted on the remaining nine policies. All nine received unanimous agreement from the expert panel and were confirmed as the final policy samples for analysis (Table 2). Among these, four policies were issued by the Central Committee of the Communist Party of China and the State Council, representing the highest level of political authority in China. The other five were issued by relevant ministries and commissions directly involved in adolescent Physical fitness promotion.

**Table 2:** Representative adolescent physical activity promotion policies in China.

**Policy ID**	**Date issued**	**Policy title**	**Issuing authority**
P1	2006.12	Decision on launching the national hundreds of millions students sunshine sports campaign	Ministry of Education, General Administration of Sport of China, Central Committee of the Communist Youth League
P2	2007.05	Opinions on strengthening adolescent physical education and improving physical fitness	Central Committee of the Communist Party of China, State Council
P3	2011.07	Regulation on ensuring 1 h of daily physical activity for primary and secondary school students	Ministry of Education
P4	2012.1	Opinions on further strengthening school physical education work	General Office of the State Council
P5	2016.05	Opinions on enhancing school physical education to promote holistic health of students	General Office of the State Council
P6	2017.11	National plan for adolescent physical activity promotion	General Administration of Sport of China, Ministry of Education, Central Civilization Office, National Development and Reform Commission, Ministry of Civil Affairs, Ministry of Finance, Central Committee of the Communist Youth League
P7	2020.08	Guiding opinions on deepening the integration of sports and education to promote adolescent health	General Administration of Sport of China, Ministry of Education
P8	2020.1	Opinions on comprehensively strengthening and improving physical education in the new era	General Office of the Central Committee of the Communist Party of China, General Office of the State Council
P9	2021.04	Notice on further strengthening the physical fitness management of primary and secondary school students	General Office of the Ministry of Education

### 3.3 Construction of the PMC-Index model

Drawing on the indicator systems used in policy evaluation by scholars such as Guo et al. (29), Yang et al. (31), and Ruiz Estrada (26), this study first established primary indicators. Based on the practical context of adolescent Physical fitness promotion in China, secondary indicators under the dimension of policy target groups (*X*5) were developed. Additionally, informed by the high-frequency terms and semantic network derived from policy text analysis, secondary indicators for policy priorities (*X*6) and policy functions (*X*7) were constructed. Following consultation with the aforementioned panel of eight experts, the final evaluation framework was established, consisting of 10 primary variables and 38 secondary variables (see Table 3).

**Table 3:** Evaluation indicators for adolescent physical activity promotion policies in China.

**Primary variable**	**Secondary variable**	**Evaluation criteria (1 = Yes, 0 = No)**	**Data source**
Policy nature (*X*_1_)	Prediction (*X*_11_)	Whether the policy includes predictive or anticipatory content	Ruiz Estrada (26)
	Recommendation (*X*_12_)	Whether the policy provides suggestions or proposals	
	Supervision (*X*_13_)	Whether the policy involves corresponding supervision mechanisms	
	Description (*X*_14_)	Whether the policy description is specific and clear	
	Orientation (*X*_15_)	Whether the policy has a clear strategic orientation	
Policy timeliness (*X*_2_)	Long-term (*X*_21_)	Whether the policy duration is more than 5 years	Ruiz Estrada (26)
	Mid-term (*X*_22_)	Whether the policy duration is between 1 and 5 years	
	Short-term (*X*_2_3)	Whether the policy duration is within 1 year	
Issuing agency (*X*_3_)	General office of CPC (*X*_31_)	Whether the issuing agency includes the General Office of the CPC Central Committee	Policy text mining
	Ministry of Education (*X*_32_)	Whether the issuing agency includes the Ministry of Education	
	General Administration of Sport (*X*_33_)	Whether the issuing agency includes the General Administration of Sport	
	Communist Youth League (*X*_34_)	Whether the issuing agency includes the Central Committee of the CYL	
	Other agencies (*X*_35_)	Whether the issuing agency includes other government institutions	
Policy instruments (*X*_4_)	Supply-based (*X*_41_)	Whether the policy involves provision of facilities, personnel, or public services	Ruiz Estrada (26)
	Environmental-based (*X*_42_)	Whether the policy includes organizational environment or cultural atmosphere building	
	Demand-based (*X*_43_)	Whether the policy encourages collaborative engagement	
Policy stakeholders (*X*_5_)	Government (*X*_51_)	Whether the policy involves participation by government authorities	Policy text mining
	Schools (*X*_52_)	Whether the policy involves participation by schools	
	Families (*X*_53_)	Whether the policy involves participation by families	
	Other organizations (*X*_54_)	Whether the policy involves other relevant organizations	
Policy focus (*X*_6_)	1-h physical training (*X*_61_)	Whether the policy emphasizes implementing daily 1-h physical exercise	Policy text mining
	Facility and faculty development(*X*_62_)	Whether the policy emphasizes improving sports venues and PE teacher development	
	Implementation of physical fitness testing standards (*X*_63_)	Whether the policy emphasizes enforcing student physical fitness standards	
	Publicity & promotion (*X*_64_)	Whether the policy emphasizes education and awareness campaigns	
	Supervision (*X*_65_)	Whether the policy emphasizes monitoring and evaluation	
	Organizational leadership (*X*_66_)	Whether the policy emphasizes working group or small team implementation	
Policy functions (*X*_7_)	Awareness improvement (*X*_71_)	Whether the policy promotes awareness of youth physical education	Policy text mining
	Social support (*X*_72_)	Whether the policy mobilizes families and communities to provide support	
	Time regulation (*X*_73_)	Whether the policy regulates daily physical activity time	
	Accountability enhancement (*X*_74_)	Whether the policy enhances supervision and accountability mechanisms	
Policy evaluation (*X*_8_)	Evidence-based (*X*_81_)	Whether the policy formulation is based on sufficient evidence	Ruiz Estrada (26)
	Clear objectives (*X*_82_)	Whether the policy objectives are clearly defined	
	Scientific planning (*X*_83_)	Whether the policy planning is scientifically feasible	
Policy support (*X*_9_)	Funding input (*X*_91_)	Whether the policy involves financial investment	Ruiz Estrada (26), policy text mining
	Organizational leadership (*X*_92_)	Whether the policy involves leadership from institutions	
	Supervision & inspection (*X*_93_)	Whether the policy involves supervision or inspection mechanisms	
	Incentives & assessment (*X*_94_)	Whether the policy involves incentive or evaluation measures	
Policy transparency (*X*_10_)	–	Whether the policy is publicly disclosed	Ruiz Estrada (26)

### 3.4 Measurement of the PMC-Index

First, each secondary variable was assigned a binary value of 0 or 1 in accordance with Equations 1, 2. Although a few previous studies have adopted differentiated scoring schemes for individual indicators, this study follows the mainstream approach of binary assignment. Second, the values of each primary variable *X*_*t*_ were calculated using Equation 3, with a possible range of 1–10. Third, based on Equation 4, the PMC-Index for each of the nine policy samples was calculated by summing the values of their primary variables. Referring to the PMC-Index rating criteria proposed by Ruiz Estrada (26), and taking into account the specific context of Physical fitness promotion policies, the evaluation results were classified into six levels (see Table 4).


(1) $$\begin{array}{l} \text{X}\sim \text{N}\left[\text{0,1}\right] \end{array}$$

(2) $$\begin{array}{l} \text{X}=\left\{\text{XR}:\left[\text{0}\sim \;\text{1}\right]\right\} \end{array}$$

(3) $$\begin{array}{l} {\text{X}}_{\text{t}}\left(\sum_{j=1}^{n}\frac{X_{tj}}{T\left(X_{tj}\right)}\right)t=\text{1, 2, 3}\ldots \infty \end{array}$$

Subsequently, according to Equation 4, sum up the scores of the primary variables of the nine sample policies to calculate the PMC-Index for China's football development policies. Meanwhile, the study divides the quality levels of the nine China's football development policies into four grades according to the policy scores: Perfect (9–10 points), Excellent (7–8.99 points), Acceptable (5–6.99 points), and Poor (0–4.99 points) (Table 4).


(4) $$\begin{array}{l} \text{PMC}=\\ \left\{\begin{array}{cccccc} X1\left(\sum_{i=1}^{5}\frac{X_{1i}}{5}\right) & + & X2\left(\sum_{j=1}^{3}\frac{X_{2j}}{3}\right) & + & X3\left(\sum_{k=1}^{3}\frac{X_{3k}}{3}\right) & +\;\\ X4\left(\sum_{l=1}^{4}\frac{X_{4l}}{4}\right) & + & X5\left(\sum_{m=1}^{5}\frac{X_{5m}}{5}\right) & + & X6\left(\sum_{n=1}^{3}\frac{X_{6n}}{3}\right) & +\;\\ X7\left(\sum_{o=1}^{3}\frac{X_{7o}}{3}\right) & + & X8\left(\sum_{p=1}^{8}\frac{X_{8p}}{8}\right) & + & X9\left(\sum_{q=1}^{3}\frac{X_{9q}}{3}\right) \end{array}\right\} \end{array}$$

**Table 4:** Rating scale for adolescent physical activity promotion policies.

**PMC-Index interval**	**[0,4)**	**[4,6)**	**[6,7)**	**[7,8)**	**[8,9)**	**9**
Policy consistency	Poor	Acceptable	Good	Excellent	Superb	Perfect
Grade code	E	D	C	B	A	A+

## 4 Results and discussion

### 4.1 PMC-Index calculation

Based on the formulas of the PMC-Index model, the scores of nine adolescent Physical fitness promotion policies in China were calculated (see Table 5). The average score across the nine policies was 6.83, all falling within the “Acceptable” level or above, indicating an overall favorable performance. Moreover, the policy quality has shown an upward trend over time, suggesting that the evolving series of policies has played a significant and positive role in promoting high-quality school physical education, deepening the integration of physical education and academic learning, and continuously enhancing students' Physical fitness and overall competencies.

**Table 5:** PMC-Index scores of adolescent physical activity promotion policies.

**ID**	**X_1_**	**X_2_**	**X_3_**	**X_4_**	**X_5_**	**X_6_**	**X_7_**	**X_8_**	**X_9_**	**Total score**	**Rank**	**Average score**	**Grade**
P1	0.60	0.33	0.60	0.67	0.50	0.70	1.00	1.00	0.30	5.70	8	0.60	D
P2	0.60	0.33	1.00	0.67	1.00	0.80	1.00	1.00	0.30	6.70	5	0.33	C
P3	0.60	0.33	0.20	0.67	0.75	0.30	0.50	1.00	0.80	5.15	9	0.73	D
P4	0.60	0.33	0.80	1.00	1.00	1.00	1.00	1.00	1.00	7.73	3	0.85	B
P5	1.00	0.33	1.00	1.00	1.00	1.00	1.00	1.00	1.00	8.13	2	0.86	A
P6	0.60	0.33	0.60	1.00	1.00	1.00	1.00	1.00	0.80	7.33	4	0.81	B
P7	0.60	0.33	0.40	1.00	0.75	0.70	0.75	1.00	0.80	6.33	7	0.92	C
P8	1.00	0.33	1.00	1.00	1.00	1.00	1.00	1.00	1.00	8.33	1	1.00	A
P9	0.67	0.33	1.00	0.67	0.75	0.80	1.00	1.00	0.50	6.65	6	0.72	C

### 4.2 Construction of the PMC-surface

This study has 10 primary variables, among which *X*10 has no secondary variables and all policy scores are 1. In view of the symmetry of the PMC matrix and the balance of the PMC surface, this item is excluded to form a 3 × 3 third-order PMC matrix Equation 5.


(5) $$PMC=\left[\begin{array}{ccc} X1 & X2 & X3\\ X4 & X5 & X6\\ X7 & X8 & X9 \end{array}\right]$$

**Figure 3:**
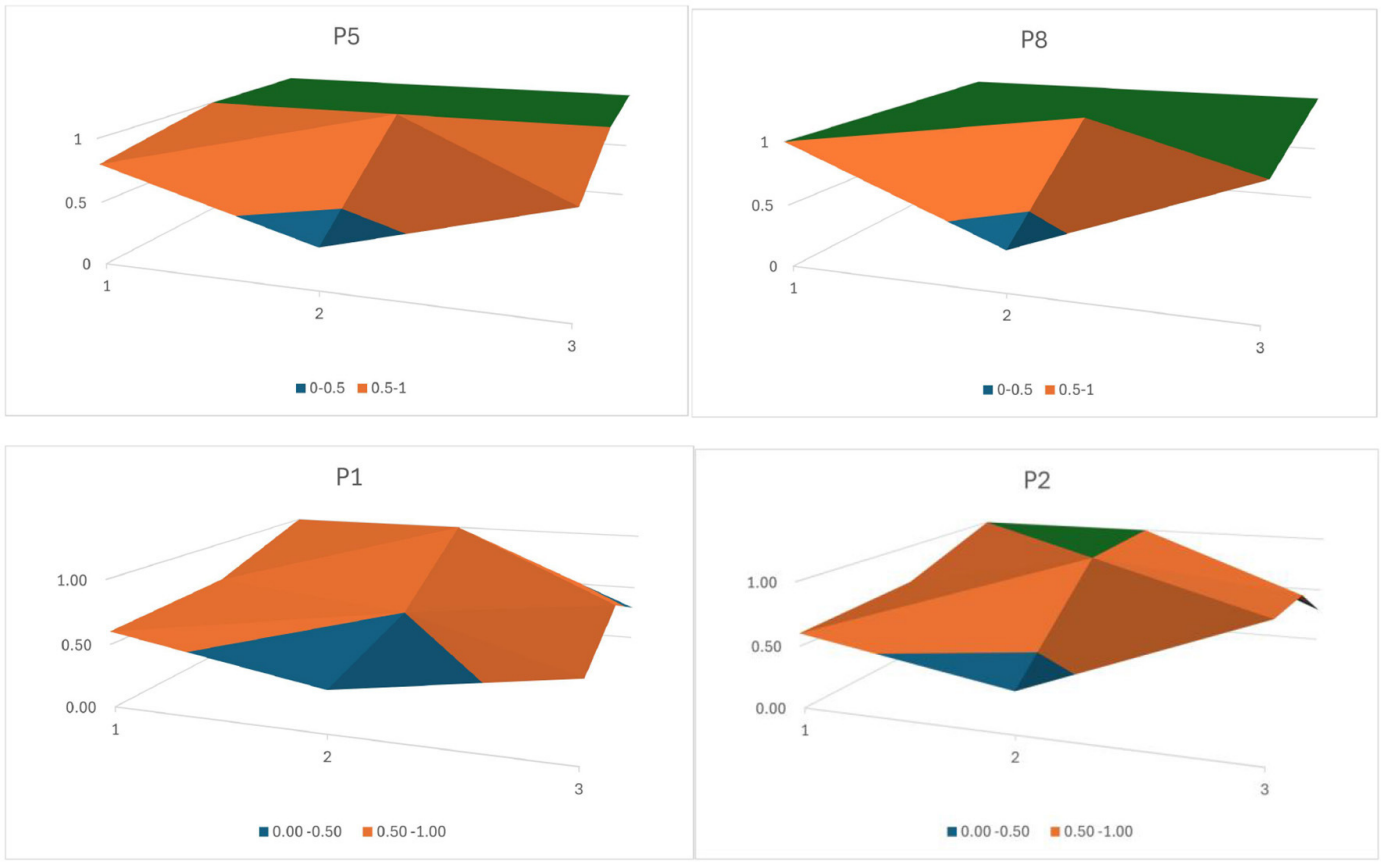
PMC-Surface diagrams of adolescent physical fitness policies.

The PMC surface plot is a visualization of the PMC-Index, which not only clearly and intuitively presents the scoring of various dimensional indicators in policy texts through its degree of concavity and average convexity-concavity but also examines the internal consistency level, structural rationality, and specific performance of individual indicators within a single policy document. Due to space constraints, this paper displays surface plots (Figure 3) of four policies with the highest, lowest, and median PMC indices. As shown, the highest-scoring P5 and P8 exhibit smooth surfaces, while P1 and P2 display noticeable concavities, indicating certain deficiencies in specific aspects.

## 5 Conclusions and implications

### 5.1 Conclusions

**Figure 4:**
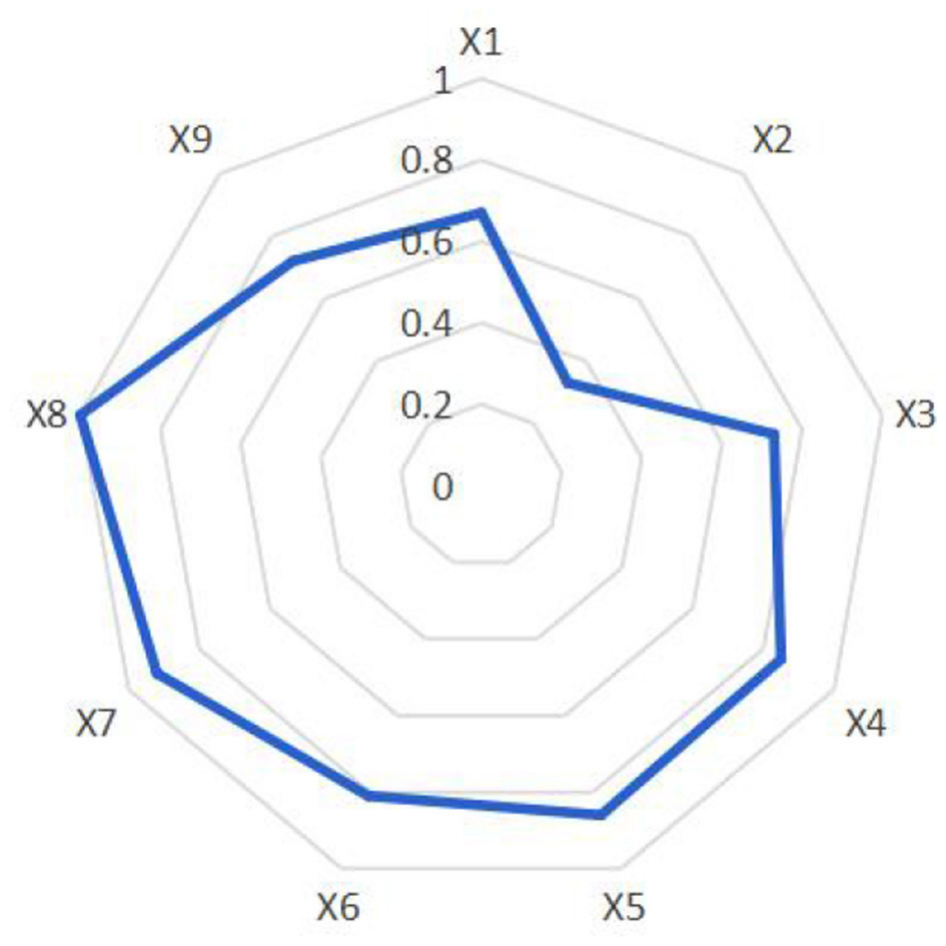
Radar map of adolescent physical fitness policy.

Based on thematic semantic network analysis and content-based text evaluation, as well as the calculated PMC-Index scores and surface plot construction, the average score across the nine adolescent Physical fitness promotion policies in China was 0.77. This indicates a high degree of rationality, scientific rigor, and effectiveness in the overall policy design and planning. The quality of policy expression was found to be relatively strong (see Figure 4).

As part of a national strategic framework, adolescent Physical fitness promotion policies in China typically follow a 5-year policy cycle, which facilitates timely adjustments in accordance with education reform and youth health monitoring data. These policies have consistently adhered to the principle of “health first” (X1), with a clear emphasis on “giving higher priority to improving students' Physical fitness” (P4). The highest-level policies concerning adolescent Physical fitness were issued by the Central Committee of the Communist Party of China and the State Council, the country's top governing bodies, which provide authoritative and strategic guidance. Sector-specific policies are mainly formulated by relevant ministries, with the Ministry of Education focusing on school-based physical education, and the General Administration of Sport aiming to enhance adolescent sports participation and competition at the societal level. Together, they form a coordinated policy structure involving central decision-making, ministerial implementation, and multi-agency collaboration including the Communist Youth League and the Central Civilization Office (*X*2, *X*3). A combination of supply-side, environment-oriented, and demand- driven policy tools has been employed to guide and regulate the development of adolescent Physical fitness promotion in China (*X*4).

The school remains the primary setting for intervention, where improved teaching staff and facilities help to strengthen external conditions. At the same time, a broader societal sports culture is cultivated to instill a scientific understanding of education, talent development, and health among youth, educators, and the general public. This supportive environment encourages the adoption of regular physical activity routines, such as 1–2 h of daily exercise (*X*6). Policies also promote collaboration between schools, sports schools, and community-based sports clubs to jointly carry out physical education, training, and competitions (*X*5). By articulating the value of physical activity in enhancing student health, the policies call for local governments and departments to implement these strategies in alignment with local conditions. Governmental and school-level awareness and commitment to Physical fitness are thus elevated. Furthermore, the policies mobilize families and society at large to support adolescent exercise initiatives, with supervision and inspection mechanisms reinforcing implementation efforts (*X*7). The policy goals (*X*8) are clearly defined, encompassing both qualitative objectives—such as “getting students onto the playground, under the sun, and into nature” (P2)—and quantitative targets, such as “ensuring that more than 85% of students engage in at least 1 h of daily exercise” (P1). Implementation is primarily secured through administrative mechanisms, including leadership organization, supervision and inspection, and performance-based evaluation and accountability measures (*X*9).

The overall performance of adolescent Physical fitness promotion policies was relatively strong. Among them, the top three policies—P8 (8.33), P5 (8.13), and P4 (7.73)—show smooth peaks in the surface plots, particularly P5 and P8. This can be attributed to several factors. First, all three were issued by the General Office of the State Council, which highlights their strategic importance. These policies feature broad coverage and clearly articulate their intentions in terms of policy rationale, objectives, guiding principles, core tasks, and implementation suggestions. Second, each of these policies demonstrates a comprehensive deployment of national-level functions. They serve as both guiding and regulatory tools by raising stakeholder awareness, encouraging social support, structuring time allocation for physical activity, and reinforcing the implementation process. Third, they place a clear emphasis on enforcement. For example, P8 specifies that local governments, education departments, and school administrators who fail to implement the policy effectively, resulting in declining Physical fitness standards or poor assessment outcomes among students, should be held accountable in accordance with legal and administrative procedures. By contrast, lower-scoring policies such as P1 (5.15) and P3 (5.70) show visible concavity in the surface plots. P1 focuses on promoting enthusiasm for physical exercise through seven proposed strategies, but it lacks sufficient policy safeguards such as dedicated funding, incentive mechanisms, or accountability frameworks. Similarly, P3 concentrates on the implementation of “1 h of daily exercise,” yet fails to address supporting aspects such as publicity and awareness, facility provision, or organizational leadership, all of which are essential for achieving the policy's intended outcomes.

### 5.2 Implications

Adolescent health and development occupy a foundational and strategic position in China. The government has continuously advanced youth Physical fitness through targeted policy measures. From 2012 to 2022, the number of physical education teachers in the compulsory education system increased from 430,000 to 700,000, representing a 62.8% growth. The proportion of schools meeting the required standards for sports field (or gymnasium) areas rose from 51% to 94.1%, while the proportion of schools with adequate sports equipment increased from 52% to 97.3%. Additionally, the proportion of adolescents with “excellent” or “good” physical fitness increased from 26.5% in 2016 to 38.5% in 2021 (37). These achievements are of great significance to the realization of the Chinese Dream and the great rejuvenation of the Chinese nation. Compared with other regions, Chinese policy texts are characterized by a “directive” style, with concise language and strong authority. Policy goals are reinforced through clearly defined quantitative targets, resource provision, and accountability mechanisms. In contrast, Western policy documents typically adopt a “discursive” style. For instance, the United States' National Youth Sports Strategy spans 112 pages and incorporates extensive data and academic references to establish policy rationale, aiming to build consensus and guide implementation across regions. The Chinese approach reflects the institutional advantage of socialism, namely the ability to “concentrate efforts on major undertakings,” and thus demonstrates high policy efficiency. To further advance adolescent Physical fitness in China, the following areas of policy optimization are recommended.

(1) Further strengthening the use of supply-oriented policy tools, with a clear focus on intervention points and quantifiable outcome targets, in order to improve the foundational conditions for Physical fitness promotion. For example, there is still a shortage of approximately 120,000 physical education teachers nationwide, particularly in rural primary and junior secondary schools, as well as at remote teaching sites. Financial investment must also continue to increase, not only to construct sports fields but also to create diverse, engaging, and creative physical activity spaces. These environments should unleash adolescents' natural vitality and creativity, making exercise more enjoyable and integrated into daily life.
(2) Issuing a dedicated a dedicated “school-family-community alliance” policy to establish an integrated coordination framework among key stakeholders. Drawing from the UK's Physical Education, School Sport and Club Links Strategy, the roles and responsibilities of schools, families, and communities should be clearly defined. A multidimensional collaboration system should be built—linking schools with communities, schools with families, and families with communities—to create a stable, substantive, and unified network for physical activity. This would expand the physical activity setting from a school-only domain to include families and communities, and help achieve the goal of 2 h of daily physical activity by 2025.
(3) Improving the quality of Physical fitness assessment and evaluation for adolescents. Although student physical fitness is assessed annually in China, the collected data is not fully utilized. A National Report on Adolescent Physical Activity and Health could be published on a regular basis. “The ‘Youth Sports and Health' Report can be published periodically, leveraging data mining and comparative analysis to generate regional ‘profiles' of physical activity promotion initiatives.” This approach facilitates a policy cycle of strategy–supervision–monitoring–evaluation–incentives/disincentives, enhancing the precision and efficiency of policy implementation and optimization. By uncovering data trends, it would also be possible to forecast shifts in adolescent Physical fitness, promoting a shift from a reactive “problem-response” model to a proactive “risk-prediction” model, thereby enhancing the foresight and resilience of public policy.

### 5.3 Limitations and further works

We emphasize that the policy evaluation method employed in this study, the PMC-Index model is scarce in the adolescent Physical fitness. To a certain extent, this research contributes to a deeper understanding of China's adolescent Physical fitness initiatives, lays a theoretical foundation for future policy development, and provides new ideas for enhancing policy design and implementation. However, this study is not without limitations due to temporal and scope-related constraints. First, the analysis is limited to national-level policies and does not include local government initiatives. For instance, the Sichuan Provincial Department of Education issued the *Notice on Ensuring a Minimum of Two Hours of Daily Physical Activity for Primary and Secondary School Students*, which implements including refining the multi-level competition system (spanning class, school, county, municipal, and provincial tiers) and establishing model schools with distinctive sports features. These initiatives effectively facilitate the implementation of the “2-h” requirement across Sichuan province, demonstrating significant research value for policy studies. Second, the selected policy samples are confined to China and do not involve cross-national comparisons. Future research can be carried out in the following directions: (1) conduct targeted analyses of adolescent Physical fitness policies issued by local governments, taking into account regional differences in economic development, educational resources, and youth growth patterns. These studies may provide practical references for bottom-up policy innovation. (2) Adopt a cross-national perspective to compare adolescent Physical fitness policies across countries. Such comparative studies may offer culturally sensitive insights for optimizing China's adolescent health policy system in line with global trends.

## Data Availability

The original contributions presented in the study are included in the article/supplementary material, further inquiries can be directed to the corresponding author.

## References

[1] Wang XL Ma C. China solution: policy supply characteristics promoting the health of adolescents through physical education. J Univ Jinan. (2024) 34:24–33. 10.20004/j.cnki.ujn.2024.03.003

[2] World Health Organization. Global Status Report on Physical Activity 2022: Executive Summary. Geneva: World Health Organization (2022).

[3] Hyde ET Omura JD Fulton JE Sarah ML Piercy KL Carlson SA. Disparities in youth sports participation in the U.S. 2017–2018. Am J Prev Med. (2020) 59:e207–10. 10.1016/j.amepre.2020.05.011 32741540

[4] Vaux-Bjerke A Polster M Fisher R McLaughlin J Staiano A. The national youth sports strategy—implementation in action. J Phys Educ Recreat Dance. (2022) 93:51–6. 10.1080/07303084.2022.2120713

[5] Pulling Kuhn A Stoepker P Dauenhauer B Castelli D Webster C Russ L. A systematic review of multi-component comprehensive school physical activity program (CSPAP) interventions. Am J Health Promot. (2021) 35:1129–49. 10.1177/08901171211013281 33955278

[6] Dowling M Washington M. Epistemic communities and knowledge-based professional networks in sport policy and governance: a case study of the Canadian sport for life leadership team. J Sport Manag. (2017) 31:133–47. 10.1123/jsm.2016-0071

[7] van Sluijs EMF Ekelund U Crochemore-Silva I Guthold R Ha A Lubans D. Physical activity behaviours in adolescence: current evidence and opportunities for intervention. Lancet. (2021) 398:429–42. 10.1016/S0140-6736(21)01259-9 34302767 PMC7612669

[8] Ferreira Silva RM Mendonca CR Azevedo VD Memon AR Silva Noll PRE Noll M. Barriers to high school and university students' physical activity: a systematic review. PLoS ONE. (2022) 17:e0265913. 10.1371/journal.pone.0265913 35377905 PMC8979430

[9] Xue E Li J. What is the value essence of “double reduction” (Shuang Jian) policy in China? A policy narrative perspective. Educ Philos Theory. (2023) 55:787–96. 10.1080/00131857.2022.2040481

[10] Zhang J Xiao WS Soh KG Yao G Mohd Anuar MAB Bai X et al. The effect of the sport education model in physical education on student learning attitude: a systematic review. BMC Public Health. (2024) 24:949. 10.1186/s12889-024-18243-0 38566018 PMC10986141

[11] Xu T Zuo F Zheng K. Parental educational expectations, academic pressure, and adolescent mental health: an empirical study based on CEPS survey data. Int J Ment Health Promot. (2024) 26:93–103. 10.32604/ijmhp.2023.043226

[12] Yue JJ Gong JL Wang JW. Predicaments and breakthroughs: Chinese student physical fitness policies based on the international action framework. J Phys Educ. (2020) 27:79–84.

[13] Cathro A Spence JC Cameron C Varela AR Morales D Kohn ER. Progress in physical activity research, policy, and surveillance in Canada: the global observatory for physical activity–GoPA! BMC Public Health. (2024) 24:2866. 10.1186/s12889-024-20322-1 39420310 PMC11487859

[14] Gao G Liu J Xu M Xia R Zhao L. A historical review of promotions of physical activity for adolescents in China from 1949 to 2020. Fron Public Health. (2024) 12:1415513. 10.3389/fpubh.2024.1415513 39668951 PMC11634797

[15] Zhang Z Zhang F. Youth sports health promotion policy since the founding of new China: retrospect and prospect. J Xian Sport Univ. (2022) 39:355–65. 10.16063/j.cnki.issn1001-747x.2022.03.013

[16] Xun CD Yang T. The evolution, logical transformation and optimization orientation of adolescent physical fitness and health policy in P.R. China. J Chengdu Sport Univ. (2023) 49:104–10. 10.15942/j.jcsu.2023.01.014

[17] Wang XZ Yang YG Kong L. Historical evolution and policy change: from “strengthening physical fitness” to “integration of sports and education”—a characteristic analysis of the evolution of physical education and health promotion policies for children and adolescents in China. China Sports Sci Technol. (2020) 56:3–10. 10.16470/j.csst.2020163

[18] Milton K Bauman A. A critical analysis of the cycles of physical activity policy in England. Int J Behav Nutr Phys Act. (2015) 12:1–9. 10.1186/s12966-015-0169-5 25638442 PMC4318185

[19] Zhang WP Wu AY Li QD. Research on the government attention to adolescent physical fitness promotion in the new era: take the policy text as an example. China Sports Sci Technol. (2023) 59:25–34+61. 10.16470/j.csst.2023052

[20] Ausenhus C Gold J M Perry CK Kozak AT Wang ML Jang SH et al. Factors impacting implementation of nutrition and physical activity policies in rural schools. BMC Public Health. (2023) 23:308. 10.1186/s12889-023-15176-y 36765324 PMC9921364

[21] Zhou T Shu WP. Difficulties in implementation of Chinese adolescent physical fitness policy based on analysis of the revised ambiguity-conflict model. J Wuhan Sports Univ. (2022) 56:44–9. 10.15930/j.cnki.wtxb.2022.02.003

[22] Resendiz E Ramírez-Varela A Mejía-Grueso J Moon J Mitáš J Brownson RC. Breaking barriers: an innovative tool to assess the national and city-level physical activity policy development to practice disconnect. J Phys Activity Health. (2024) 21:425–33. 10.1123/jpah.2023-0471 38242113

[23] Alghannam AF Malkin JD Al-Hazzaa HM AlAhmed R Evenson KR Rakic S. Public policies to increase physical activity and reduce sedentary behavior: a narrative synthesis of “reviews of reviews”. Glob Health Action. (2023) 16:2194715. 10.1080/16549716.2023.2194715 37021717 PMC10081086

[24] Zheng X Dong Y. Review on quantitative analysis of political texts. J Modern Inf . (2021) 41:168–77.

[25] Fang SY Liu Q. A review on application of PMC-index model in policy documents quantitative research. J Modern Inf . (2024) 44:168–76.

[26] Ruiz Estrada MA. Policy modeling: definition, classification and evaluation. J Policy Model. (2011) 33:523–36. 10.1016/j.jpolmod.2011.02.003

[27] Li G Lv LJ Wang YC. Quantitative evaluation of science education policies in primary and secondary schools in china during the “14th five-year plan” period—analysis based on PMC-index model. J Northeast Norm Univ. (2024) 4:59–72. 10.16164/j.cnki.22-1062/c.2024.04.007

[28] Zhang Q Ma HQ Niu XH. Research on Chinese data security policy evaluation based on PMC-index model. J Mod Inf . (2024) 44:13–27+136.

[29] Guo D Qi L Song X. Quantitative evaluation of the medicine innovation policy in China: based on the PMC-Index model. Front Public Health. (2024) 12:1403320. 10.3389/fpubh.2024.1403320 38818446 PMC11137217

[30] Zhang YX Tian KY Yu XY Jia YH Hong LX. Evaluation of TCM medical insurance support policy based on PMC-index model. Chin Health Serv Manag. (2025) 42: 51–6+100.

[31] Yang C Yin S Cui D Mao Z Sun Y Jia C. Quantitative evaluation of traditional Chinese medicine development policy: a PMC-Index model approach. Front Public Health. (2022) 10:1041528. 10.3389/fpubh.2022.1041528 36466538 PMC9715970

[32] Yue C Zhu PJ Peng H Ying L Lei C. Quantitative evaluation of clinical research policies in China based on PMC-Index model. Chin J Evid Based Med. (2024) 24:666–72.

[33] Lang ZH Wu YX Zhou LF Xu LF Yu QQ. Quantitative evaluation of high-quality development policies of public hospitals at provincial level based on PMC-index model. Chin Hosp Manag. (2024) 44:1–4+9.

[34] Liao WZ Ma CY Li XM. Analysis of healthy China initiative policies based on PMC-index model. Chin Hosp Manag. (2024) 44:23–7.

[35] Chen J Gao Y Wang X. Evaluation of China's fertility policy based on PMC modeling. Front Public Health. (2025) 13:1533307. 10.3389/fpubh.2025.1533307 39975774 PMC11835804

[36] Liu Y Jiao M Wang Y Ma A. Quantitative evaluation of China's public health emergencies response policies: a PMC-Index model approach. BMC Public Health. (2025) 25:266. 10.1186/s12889-024-21180-7 39844109 PMC11752722

[37] Ministry of Education of the People's Republic of China. The Number of Physical Education Teachers in Compulsory Education Increased to 700,000. China Education Online. Beijing: Ministry of Education (2023).

